# Applied multimodal diagnostics in a case of presenile dementia

**DOI:** 10.1186/s12883-016-0647-7

**Published:** 2016-08-09

**Authors:** Sonja Schönecker, Matthias Brendel, Marion Huber, Christian Vollmar, Hans-Juergen Huppertz, Stefan Teipel, Nobuyuki Okamura, Johannes Levin, Axel Rominger, Adrian Danek

**Affiliations:** 1 Department of Neurology, Ludwig-Maximilians University, Munich, Germany; 2 Department of Nuclear Medicine, Ludwig-Maximilians University, Munich, Germany; 3 Swiss Epilepsy Center, Zurich, Switzerland; 4 Department of Psychosomatic Medicine, University of Rostock, Rostock, Germany; 5 German Center for Neurodegenerative Diseases, Rostock, Germany; 6 Department of Pharmacology, Tohoku University School of Medicine, Sendai, Japan; 7 German Center for Neurodegenerative Diseases, Munich, Germany

**Keywords:** Case report, Alzheimer’s disease, Biomarkers, CSF, MRI, FDG-PET, tau-PET, Amyloid-PET

## Abstract

**Background:**

Alzheimer’s disease (AD) is the most common cause of dementia in the elderly. The possibility of disease-modifying strategies has evoked a need for early and accurate diagnosis. To improve the accuracy of the clinical diagnosis of AD, biomarkers like cerebrospinal fluid (CSF) and neuroimaging techniques like magnetic resonance imaging (MRI) and positron emission tomography (PET) have been incorporated into the diagnostic guidelines of AD.

**Case presentation:**

In this case report we outline in reference to one of our patients with presenile dementia the current approaches to the diagnosis of AD. The patient was a 59-year old woman presenting with progressive memory decline. CSF-Aβ_42_ was normal while P-tau was slightly increased. FDG-PET indicated a pattern typical for AD, amyloid-PET showed an extensive global amyloid load, and tau-PET depicted a pronounced hippocampal tracer accumulation. The MRI scan was rated as normal at routine diagnostics, however quantitative volumetric analysis revealed significant atrophy especially of the parietal lobe. The combination of biomarkers and neuroimaging techniques was therefore suggestive of an underlying AD pathology.

**Conclusions:**

To enable early and accurate diagnosis of AD and thereby also patient recruitment for anti-tau or anti-β-amyloid therapeutic trials, a combination of biomarkers and neuroimaging techniques seems useful.

## Background

Alzheimer’s disease (AD) is the most common cause of dementia and is estimated to affect 106.8 million people worldwide by the year 2050 [1]. It is clinically characterized by progressive memory and language impairment, functional and behavioural disturbances and visuospatial deficits [2]. A definite diagnosis of AD still relies on post-mortem histopathological detection of intracellular neurofibrillary tangles and extracellular amyloid plaques [3]. It is widely acknowledged that histopathological changes start years before clinical manifestation of the disease [4]. As first disease modifying therapies are approaching early and accurate diagnosis of AD becomes increasingly important [5]. Therefore the National Institute on Aging and the Alzheimer’s Association have revised the criteria for the diagnosis of AD [6]. To assess the probability of an underlying AD pathology biomarkers of the disease and neuroimaging techniques have been incorporated into the diagnostic guidelines of AD. Here we present an exemplary case of presenile dementia based on which we discuss the approaches to the diagnosis of AD.

## Case presentation

A 59 year old retired attorney, with 18 years of education presented with a three year history of progressive memory decline. For about one year she hadn’t been able to do the shopping or the cooking. No behavioural changes, language impairment, severe fluctuations of attention and alertness, recurrent visual hallucinations or history of repetitive brain trauma were reported. Family history was positive, her mother as well as an uncle had developed late-onset dementia.

The Mini-Mental-State Examination which had been performed three years ago because of subjective memory impairment had been rated as normal. For neuropsychological testing we applied the CERAD plus battery additionally including Trail Making Test A and B as well as verbal fluency tests. The scores of almost all domains of the CERAD plus battery were at least −1.37 standard deviations below the age- and education-adjusted norm values. She scored 24 out of 30 points in the Mini-Mental-State Examination. Naming (Boston-Naming-test) was intact whereas phonemic verbal fluency was slightly (11 words in 1 min) and semantic fluency (9 words in 1 min) was highly reduced. Memory was highly impaired. She displayed intrusions and showed a reduced performance of word list recall and recognition. Constructional practice was impaired as well. Results of the Trail-Making Test showed reduced visual attention as well as reduced speed of processing. Cognitive flexibility measured by Trail-Making-Test B was also poor.

At first presentation the neurological examination was normal. In particular no manifest or latent paresis and no sensory deficit could be detected. Deep tendon reflexes were mildly hypoactive without any pathological reflexes. Cranial nerves were also intact. There was no evidence of extrapyramidal features.

Laboratory tests for metabolic causes of dementia, for example for vitamin B_12_, thyroid, liver and renal function, thiamine level and folate were within the normal range. P-tau (66 pg/ml, N < 61 pg/ml) in cerebrospinal fluid (CSF) was only slightly increased, whereas total Tau (293 pg/ml, N < 500 pg/ml) and Aβ_42_ (1171 pg/ml, N > 500 pg/ml) were normal. All other CSF parameters were within the normal range.

**Fig. 1:**
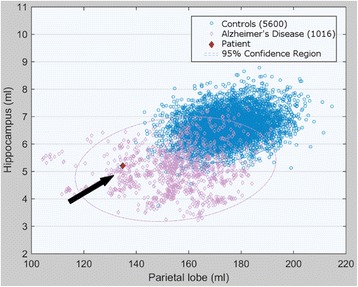
Volumetric MRI analysis of our patient’s (dark arrow) hippocampus and parietal lobe compared to AD patients and age-matched healthy controls after intracranial-volume correction. Z-scores of hippocampal and parietal lobe volume compared to healthy controls were −2.9 and −4.2 respectively. Our patient’s datapoint lay within the 95 % confidence region of Alzheimer’s disease and outside the 95 % confidence region of healthy controls and was therefore automatically assigned to the Alzheimer’s disease group

The MRI scan which had been performed three years ago as well as the MRI scan at first presentation were interpreted as normal at visual inspection. Especially no sign of global or regional atrophy could be detected. However, atlas-based volumetric MRI analysis [7, 8] showed a significant reduction especially of temporal and parietal lobe volumes. Z-scores for the hippocampal and parietal volumes compared to healthy controls were −2.9 and −4.2 respectively (Fig. 1).

**Fig. 2:**
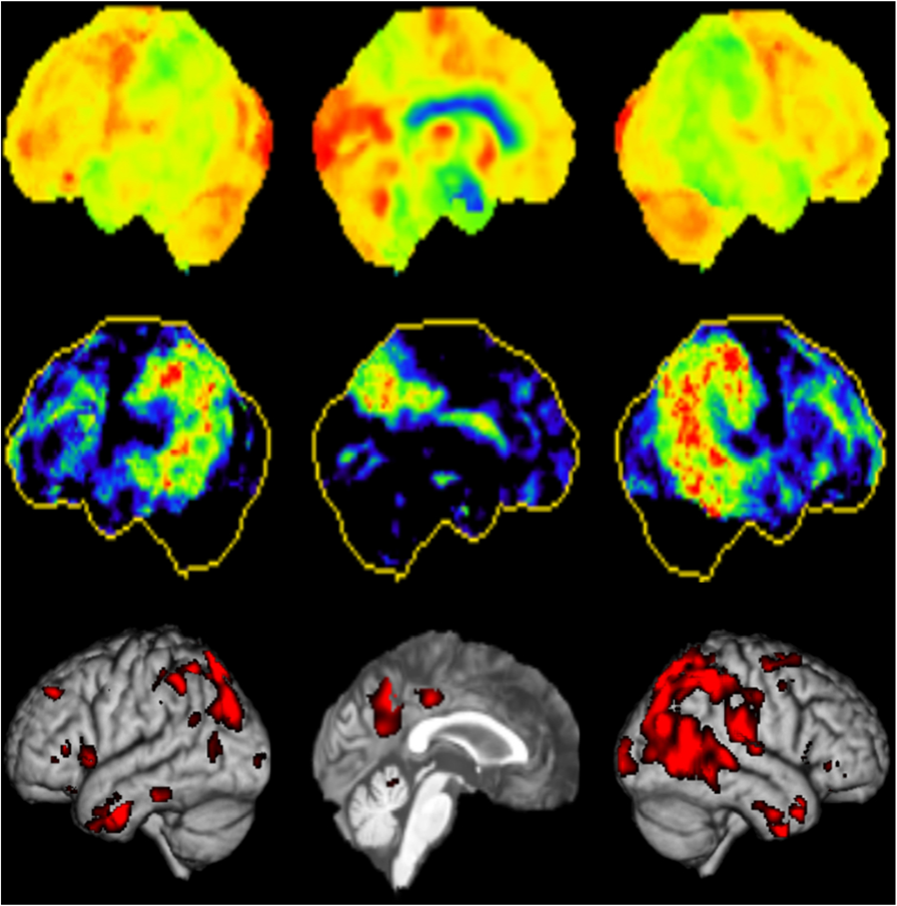
Positron emission tomography of the brain with F-18-labeled fluorodeoxyglucose (FDG-PET) and three-dimensional surface representation of brain regions with grey matter reduction of our 59-year-old AD patient compared to 18 healthy controls. Top: regional uptake of radiolabelled glucose; the left lateral, left medial and right lateral views of the brain are presented. Middle: regional metabolic reduction compared to age-matched healthy controls according to the methodology of Minoshima et al. [53]. There is an asymmetric metabolic reduction parietotemporal and in posterior cingulate cortex with a more pronounced reduction of glucose metabolism in the right hemisphere. Bottom: color-coded brain matter reduction. Z-values of grey matter reduction are projected on T1-weighted, averaged brain surface of healthy control subjects

Moreover, a quantitative analysis based on voxel-wise z-score analysis of patient grey matter segments and grey matter segments from an age and sex-matched control sample after bias correction and spatial normalization in a common standard space following an established method [9] showed a slight asymmetry with more pronounced atrophy of rightsided parietotemporal cortical areas (voxel based grey matter reduction z > −1.96) (Fig. 2). It has to be noted that for the quantitative analysis, data of the comparison group came from another MRI scanner than the scan of the patient. Acquisition parameters were harmonized between scanners to accommodate possible scanner effects.

Fluorodeoxyglucose-PET (FDG-PET) showed an asymmetric reduction of brain glucose metabolism of parietal and temporal cortical areas with a more pronounced reduction of glucose metabolism in the right hemisphere. In addition glucose metabolism of the posterior cingulate cortex was significantly reduced (Fig. 2).

**Fig. 3:**
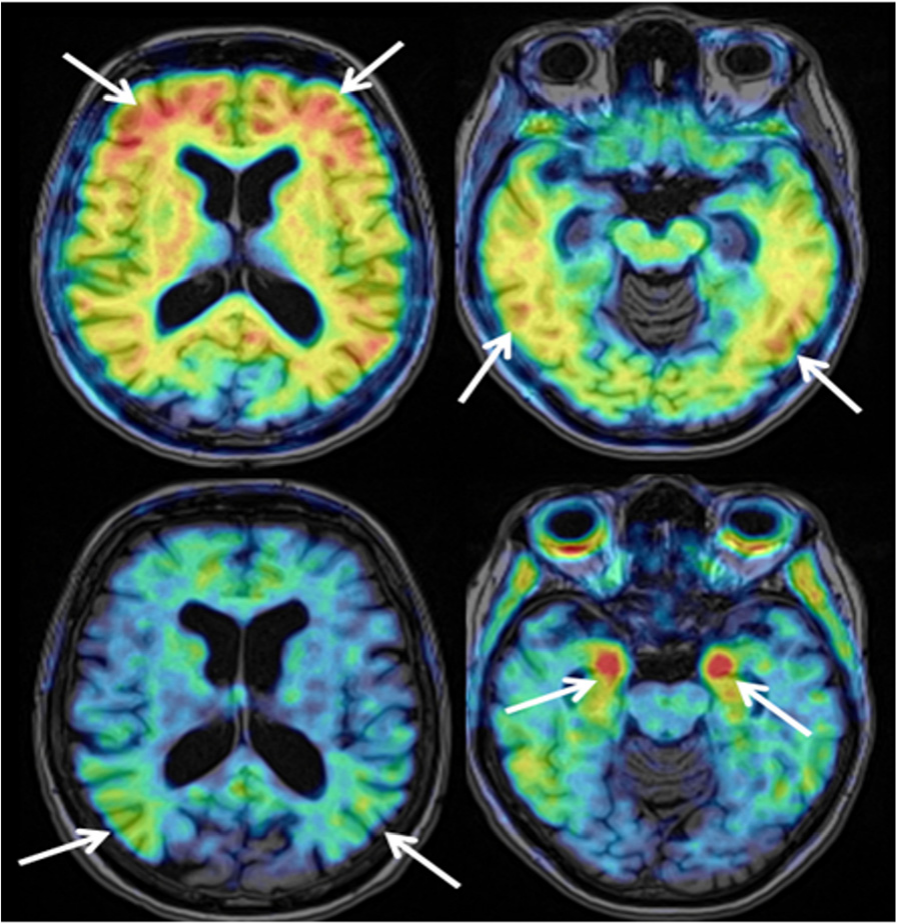
Aβ and tau imaging with FBB (top) and THK-5351 (bottom) of our 59 year old patient with Alzheimer’s disease. FBB and THK-5351 binding patterns demonstrate the different distributions of Aβ and tau deposits in the brain of our Alzheimer’s disease patient. FBB-PET scan revealed a massive global amyloid load with a pronounced FBB retention in parietotemporal, frontal and posterior cingulate/precuneal cortices. In contrast THK-5351 retention was markedly elevated in the hippocampus, whereas onlya slightly increased THK-5351 retention was observed in parietotemporal cortical areas

The patient underwent a florbetaben-PET (FBB-PET) scan which showed extensive FBB retention that was greater in parietotemporal, frontal and posterior cingulate/precuneus cortex and less pronounced in the occipital cortex. Basal ganglia were also slightly affected (Fig. 3).

The tau-PET scan with THK-5351 on the other hand showed high tracer retention in both hippocampi as well as moderately increased tracer retention in parietotemporal cortical areas. There was no elevated tracer retention in other cortical areas (Fig. 3).

## Conclusions

As our patient presented with a three year history of cognitive decline that interfered with activities of daily living and scored low in almost all domains of the CERAD plus battery a clinical diagnosis of AD was made [6]. Because of the presenile age at onset and the positive family history a monogenic form of AD might be possible. Genetic testing could have excluded conditions mimicking sporadic AD-like dementia. Our patient however refused genetic testing. Sensitivity and specificity of the clinical diagnosis of AD compared to post-mortem histopathological diagnosis ranges from 70.9 to 87.3 % and from 44.3 to 70.8 % respectively [10]. The combination of biomarkers and neuroimaging techniques was suggestive of an underlying AD pathology. In absence of a neuropathological confirmation of AD pathology, a definite diagnosis of AD, however, was not possible. The most important alternative diagnosis in a case of presenile dementia are dementia with Lewy bodies (DLB), frontotemporal dementia with memory impairment (FTD) and chronic traumatic encephalopathy (CTE). As our patient had no pronounced variation in attention and alertness, reported no recurrent visual hallucinations and displayed no features of parkinsonism the core features of DLB were not fulfilled [11]. Furthermore no progressive deterioration of personality or social comportment was reported and there was no evidence of progressive language impairment. Therefore there was no clinical evidence for behavioural variant frontotemporal dementia [12] or primary progressive aphasia [13]. As there was no history of recurrent impacts to the head or body, CTE seemed unlikely as well [14]. Table 1 summarizes biomarker and neuroimaging findings in autopsy confirmed cases of DLB, FTD and CTE. [References in Table 1: [15–30]]. In the following the current diagnostic approaches used in the diagnosis of AD are illustrated with reference to our 59-year old patient.

**Table 1:** Summary of biomarker and neuroimaging findings in autopsy confirmed DLB, FTD and CTE cases

	Population	CSF biomarker/Ligand	Major findings
CSF			
Clark et al.(15]	60 AD, 10 FTD, 3 DLB	total Tau, Aβ42	higher total tau in AD compared to FTD and DLB
			Aβ42 reduced in AD compared to FTD but not compared to DLB
Slaets et al. [16]	30 AD, 13 DLB with SP, 5 DLB without SP 30 AD, 9 DLB with NFT, 9 DLB without NFT	P-Tau, total Tau, Aβ42	Aβ42 reduced in AD and DLB with SP compared to DLB without SP no difference in Aβ42 levels of AD and DLB with SP patients
		P-Tau, total Tau, Aβ42	P-Tau higher in AD compared to DLB with and without NFT no difference in P-Tau levels of DLB with and without NFT no difference of total Tau between the DLB subgroups and AD
Koopmann et al. [17]	95 AD, 18 DLB, 10 FTD	P-Tau, total Tau, Aβ42	P-Tau cut-off for differentiating AD from FTD 35.3 pg/ml, from DLB 52.8 pg/ml total Tau level: AD > DLB > FTD Aβ42 level: AD < DLB = FTD
Bian et al. [18]	AD 19, FTD 30	total Tau, Aβ42	total Tau and tau/Aβ42 ratio lower in FTD than in AD
Toledo et al. [19]	71 AD, 29 FTD	P-Tau, total Tau, Aβ42	high sensitivity and specificity of combined CSF biomarkers in classifying AD against FTD P-Tau and total Tau higher in AD compared to FTD Aβ42 lower in AD compared to FTD
MRI			
Vemuri et al. [20]	48 AD, 47 FTD, 20 DLB		atrophy pattern in AD: temporoparietal association cortices and medial temporal lobe FTD: frontal and temporal lobes DLB: bilateral amygdalae, dorsal midbrain, inferior temporal lobe
Rabinovici et al. [21]	11 AD, 18 FTD		atrophy in AD: posterior temporoparietal and occipital atrophy atrophy in FTD: medial prefrontal and medial temporal cortex, insula, hippocampus, amygdala
Burton et al. [22]	11 AD, 23 DLB		pronounced medial temporal lobe atrophy in AD compared to DLB patients
Kantarci et al. [23]	2 AD, 3DLB		more pronounced hippocampal atrophy in AD compared to DLB
McKee et al. [24]	1 CTE		generalized cortical atrophy, enlargement of ventricles, cavum septum pellucidum
FDG-PET			
Minoshima et al. [25]	10 AD, 4 DLB		AD and DLB: hypometabolism in posterior cingulate, parietotemporal and frontal association cortices additional occipital hypometabolism in DLB
Albin et al. [26]	3 AD-DLB, 3 DLB		compared to AD additional hypometabolism in occipital association and primary visual cortex
Kantarci et al. [23]	2 AD, 3 DLB		low occipital FDG-uptake in 1 AD patient and all DLB patients
Foster et al. [27]	31 AD, 14 FTD		AD: temporoparietal and posterior cingulate hypometablism FTD: frontal, anterior cingulate and anterior temporal hypometabolism
Amyloid-PET			
Kantarci et al. [23]	2 AD, 3 DLB	PiB	high global cortical PiB retention in one AD patient, low global cortical PiB in the other
			2 DLB patients with borderline PiB retention, 1 DLB patient with high PiB retention
Bacskai et al. [28]	1 DLB	PiB	tracer uptake in posterior cingulate, precuneus, posterior parietal, middle and inferior temporal, insular, lateral and orbital frontal cortices
Rabinovici et al. [29]	3 AD, 7 FTD	PiB	higher PiB retention in AD compared to FTD better classification accuracy of PiB-PET compared to FDG-PET
Tau-PET			
Ghetti et al. [30]	1 FTD	T807	elevated tracer uptake in anterior, temporal and parietal cortex as well as basal ganglia

*CSF* cerebrospinal fluid, *AD* Alzheimer’s disease, *FTD* frontotemporal dementia, *DLB* dementia with lewy bodies, *CTE* chronic traumatic encephalopathy, *SP* senile plaque, *NFT* neurofibrillary tangles, *MRI* magnetic resonance imaging, *PET* positron emission tomography, *FDG* fluorodeoxyglucose

Neuropsychological testing is commonly used as an aid in diagnosing AD. In our patient the scores of almost all domains of the CERAD plus battery were well below average. Memory was most severely affected. As the earliest pathological changes in AD occur in medial temporal lobe structures [31] episodic memory is usually the first cognitive ability to decline, typically followed, like in our case by additional deficits in language and semantic knowledge, executive functions, working memory, attention and visuospatial abilities [32].

Results of laboratory testing for example for vitamin B_12,_ thyroid function, thiamine level and folate, were within the normal range. Laboratory testing should be performed in every AD patient to rule out metabolic causes of dementia.

CSF did not suggest malignancy, neuroinflammation or infection. P-tau that reflects the intensity of neuronal degeneration was slightly increased. Interestingly, although amyloid-PET revealed an extensive global amyloid load, CSF-Aβ_42_ on the other hand was normal. It has been shown that amyloid load at autopsy of AD patients is inversely correlated to CSF-Aβ_42_ whereas tau load is positively correlated to CSF P-tau and total tau [33]. Yet although CSF and amyloid-PET measurements of Aβ_42_ are consistent in the majority of patients a dissociation between Aβ_42_ measurements is not uncommon [34]. Especially the combination of CSF biomarkers may increase diagnostic certainty of AD pathology. Yet, as CSF biomarkers do not change during the clinical phase of AD, they cannot be used as markers of disease progression [35]. In comparison to AD patients, DLB and FTD patients present significantly lower P-tau and total tau levels [17]. There is however some overlap in CSF P-tau and total tau between AD, DLB and FTD patients. Aβ_42_ is generally decreased in DLB. Most studies could not define valuable CSF Aβ_42_ cut-off scores for differentiation of DLB from AD [36]. CSF Aβ_42_ levels of FTD patients on the other hand are significantly higher compared to AD patients [18]. The combination of CSF P-tau, total tau and Aβ_42_ has shown high diagnostic sensitivity and specificity in distinguishing AD from DLB and FTD patients. In CTE one would expect normal CSF Aβ_42_ levels and an elevated P-tau/total tau ratio [14]. Large biomarker studies on autopsy confirmed CTE cases however are lacking. AS CSF P-tau was only slightly increased while CSF total tau and Aβ_42_ were within the normal range, CSF analysis was not suggestive of an underlying AD pathology in our case.

The MRI scan was interpreted as normal at routine diagnostics, especially no sign of normal-pressure hydrocephalus, cerebrovascular disease, tumours or regional/global atrophy could be detected. Volumetric MRI analysis however revealed significant atrophy of the parietal and temporal lobe, especially of the hippocampus, and may therefore be useful to obtain rater- independent and objective results [7, 8]. Comparison of atrophy patterns can differentiate AD patients with a high sensitivity and specificity from healthy controls and other dementia syndromes like DLB and FTD [20]. In DLB significant gray matter loss is detectable in bilateral amygdalae, the middle temporal lobe as well as the dorsal ponto-mesencephalic junction area [20]. In FTD patients atrophy is normally restricted to the frontal and temporal lobes, with relative sparing of the parietal and occipital lobes. MRI may also be useful to detect neuropathological changes observed in CTE like whole brain atrophy or cavum septum pellucidum with occasional fenestration [24]. In our case volumetric MRI analysis showed a typical AD atrophy pattern with pronounced atrophy of the parietal and temporal lobe, especially the hippocampus, and therefore supported the diagnosis of AD. In Alzheimer’s disease patients, especially hippocampal atrophy (Fig. 1) seems to be highly correlated with episodic memory impairment [37].

In the FDG-PET scan (Fig. 2) the typical AD pattern consisting of a reduction of cerebral glucose metabolism in precuneus, posterior cingulate and parietotemporal association cortices [38] could be detected. In comparison, DLB patients show an additional significant metabolic reduction in the occipital cortex, particularly the primary visual cortex, which distinguishes DLB with a high sensitivity and specificity from AD patients [25]. FTD on the other hand causes hypometabolism in the frontal lobes, the anterior temporal cortex and anterior cingulate cortex [27]. FDG-PET studies that evaluated glucose metabolism in subjects with repetitive brain trauma have shown inconsistent findings [39, 40]. In summary, FDG-PET clearly supported AD as the most probable diagnosis in our case. Like structural MRI, FDG-PET represents a marker of neuronal injury. Retrospective investigations showed a sensitivity of 84 % and a specificity of 74 % for FDG-PET in predicting post-mortem AD pathology at autopsy [41]. FDG-PET thereby outperformed the initial clinical evaluation. Furthermore, FDG-PET seems to be a suitable predictor of conversion to AD in patients with mild cognitive impairment [42].

Amyloid- and tau-Pet represent new diagnostic tools. To present a definite diagnosis of AD relies on post-mortem histopathology. Yet these new imaging techniques permit non-invasive visualization and quantification of the two histological hallmarks of the disease.

Amyloid-PET showed a characteristic increase of tracer uptake in cortical regions known to have a high amount of amyloid burden in AD, i.e. frontal, parietal and lateral temporal cortex (Fig. 3). DLB patients can show a similar pattern yet with lower amount of Aβ ligand binding compared with AD patients [43]. FTD patients on the other hand display low cortical tracer retention such that amyloid PET has shown high accuracy in discriminating AD from FTD [44, 45]. Variable degrees of diffuse β-amyloid can be detected in about 47 % of autopsy confirmed CTE cases [46]. Amyloid-PET may therefore differentiate between CTE and AD by identifying different amyloid-deposition patterns. Further studies are needed to determine the topography of β-amyloid depositions in CTE. Showing a high amount of tracer uptake in parietotemporal, frontal and posterior cingulate/precuneus cortex, FBB-PET was suggestive for AD in our case. Recent phase III studies in which the in-vivo uptake of 18-F-labelled amyloid tracers was compared to post-mortem amyloid load showed a sensitivity and specificity of 88 to 92 %, and 88 to 100 % respectively for the detection of amyloid deposits in AD patients [47–49]. As amyloid deposition probably represents a very early event in the course of the disease that occurs years before onset of dementia symptoms, amyloid-PET may allow early and even presymptomatic diagnosis [4].

Increased tau-tracer retention could be detected in parietotemporal cortical areas, especially in the hippocampus (Fig. 3). In contrast to amyloid-PET the sensitivity and specificity of tau-PET imaging have yet to be determined. Post-mortem studies have shown that the amount of tau deposition is highly related to the severity of dementia [50]. In addition to aiding in the early and differential diagnosis of Alzheimer’s disease tau-PET may therefore serve as a marker of disease progression. Recently, first experiences with tau-PET in DLB were reported [51]. Tau deposition is elevated in some cases of DLB, especially in the inferior temporal region. Tau-PET scans may be positive in some variants of FTD. A first case report of a P301L *MAPT* mutation carrier showing elevated tau tracer uptake in frontal, anterior temporal and parietal cortex as well as in basal ganglia has been published [30]. Hitherto a single case report of a patient with a clinical diagnosis of CTE who underwent tau-PET imaging has been published [52]. Increased tracer uptake could be detected in globus pallidus, putamen, hippocampus and substantia nigra. Because of increased tracer retention in basal ganglia the detected distribution of tracer retention seemed more suggestive of progressive supranuclear palsy. However the patient did not manifest the typical clinical symptoms of progressive supranuclear palsy. In summary tau-PET also supported a diagnosis of AD in our case.

Overall, although CSF Aβ_42_ was normal, the combination of biomarker and neuroimaging findings in this case was still suggestive of AD pathology. Table 2 rates the biomarker and neuroimaging findings of our case with respect to possible differential diagnosis.

**Table 2:** Rating of biomarker and neuroimaging findings of our case with respect to possible differential diagnosis

Biomarker/neuroimaging findings	AD	FTD	DLB	CTE
CSF P-tau	+	-	-	-
CSF Aβ42	-	O	-	O
MRI - clinical routine	O	O	O	O
MRI - voxel-based	++	-	+	-
FDG-PET	++	-	O	-
amyloid-PET	++	-	+	+
tau-PET	++	O	O	O

++ highly increases probability, + increases probability, O probability unchanged, − decreases probability

As some of the illustrated diagnostic approaches provided converging evidence, e.g. CSF P-tau, MRI, FDG-PET and Tau-PET each indicated an AD typical neuronal degeneration and thereby provided somewhat redundant information, the necessity for a diagnostic algorithm becomes obvious. A sequential diagnostic process where widely available diagnostic tools like neuropsychological testing to establish the diagnosis of a dementia syndrome and laboratory testing to exclude metabolic causes of dementia are performed in a first step may be useful. Such a baseline testing could help to select patients that profit from further diagnostic work-up. In a second step CSF examination and structural MRI may be rational, on the one hand to further exclude potentially treatable causes of dementia like neuroinflammation or normal pressure hydrocephalus and on the other hand to obtain evidence for an AD-related pathological process and AD typical neuronal degeneration. In patients presenting with an atypical clinical course or atypically early age of onset the more expensive nuclear medicine diagnostic techniques FDG-, amyloid- and tau-PET might be useful to differentiate between AD and important differential diagnoses like pseudo-dementia or frontotemporal dementia. They may also serve as markers of disease progression and prognostic markers. Especially tau and amyloid imaging may furthermore be useful for patient recruitment and serve as a surrogate marker for monitoring the efficacy of future anti-tau or anti-amyloid strategies.

## Abbreviations

AD, Alzheimer’s disease; CSF, cerebrospinal fluid; CTE, chronic traumatic encephalopathy; DLB, dementia with lewy bodies; FBB, florbetaben; FDG, fluorodeoxyglucose; FTD, frontotemporal dementia; MRI, magnetic resonance imaging; PET, positron emission tomography
